# Exposure to a nocturnal light pulse simultaneously and differentially affects stridulation and locomotion behaviors in crickets

**DOI:** 10.3389/fphys.2023.1151570

**Published:** 2023-03-16

**Authors:** Keren Levy, Anat Barnea, Amir Ayali

**Affiliations:** ^1^ School of Zoology, Tel Aviv University, Tel-Aviv, Israel; ^2^ Department of Natural and Life Sciences, The Open University of Israel, Ra’anana, Israel; ^3^ Sagol School of Neuroscience, Tel Aviv University, Tel-Aviv, Israel

**Keywords:** artificial light at night, ALAN, light pollution, masking, insect, circadian rhythm, *Gryllus bimaculatus*

## Abstract

It is crucial for living organisms to be in synchrony with their environment and to anticipate circadian and annual changes. The circadian clock is responsible for entraining organisms’ activity to the day-night rhythmicity. Artificial light at night (ALAN) was shown to obstruct the natural light cycle, leading to desynchronized behavioral patterns. Our knowledge of the mechanisms behind these adverse effects of ALAN, however, is far from complete. Here we monitored the stridulation and locomotion behavior of male field crickets (*Gryllus bimaculatus*), raised under light:dark conditions, before, during, and after exposure to a nocturnal 3-h pulse of different ALAN intensities. The experimental insects were then placed under a constant light regime (of different intensities); their behavior was continuously monitored; and the period of their daily activity rhythms was calculated. The light pulse treatment induced a simultaneous negative (suppressing stridulation) and positive (inducing locomotion) effect, manifested in significant changes in the average level of the specific activity on the night of the pulse compared to the preceding and the following nights. The transition to constant light conditions led to significant changes in the period of the circadian rhythms. Both effects were light-intensity-dependent, indicating the importance of dark nights for both individual and population synchronization.

## 1 Introduction

The entrainment of a living organism’s circadian clock is mostly accomplished by means of synchronization to light (Aschoff, 1960; Saunders et al., 2002; Merrow et al., 2005; Tomioka and Matsumoto, 2019; Helfrich-Förster, 2020), which is the most reliable environmental signal of the Earth’s cycle. Most animals depend on perceiving the light’s rhythmicity for the anticipation and regulation of their activities: for choosing their foraging and sleep times, and for predator avoidance, as well as for the regulation of internal, hormonal, and molecular processes (Saunders et al., 2002; Kronfeld-Schor et al., 2013; Saunders, 2013; Kuhlman et al., 2018). In accordance with the species’ diurnal, nocturnal, or crepuscular nature, the same input may trigger different internal, and behavioral reactions (Mrosovsky, 1999; Helm et al., 2017).

Any obstruction of the light-dark cycle (or its perception) may affect daily activity rhythms, manifested in either entrainment to the new conditions, free-running behavior (an endogenous internal rhythm, that is not synchronized to the environment), or arrhythmic behavior. Yet another effect may be that of masking (see Aschoff, 1960; Mrosovsky, 1999; Kronfeld-Schor et al., 2017): i.e., an instantaneous reaction to an external signal that does not entrain the rhythm but overrides the circadian clock circuits. Such triggered interference may lead to an increase in activity (i.e., positive masking) or to a decrease in activity (i.e., negative masking), depending on the threshold and susceptibility of the relevant species and specific behavior to the stimuli (Mrosovsky, 1999). For example, light may trigger activity in diurnal species while eliciting an urge to rest in nocturnal species (Helm et al., 2017), a phenomenon that has been described in various species (Aschoff and von Goetz, 1988; Germ and Tomioka, 1998; Cohen et al., 2010; Rotics et al., 2011; Erkert et al., 2012; Spoelstra et al., 2018).

An important and constantly increasing source of obstruction of the light-dark cycle is that of artificial light at night (Hölker et al., 2010; Falchi et al., 2016; Falchi et al., 2019). ALAN reduces (even eliminates) the nocturnal darkness and thus disturbs the circadian entrainment to the daily periodicity. It modifies the time, duration, and intensity of the surrounding lighting conditions, as well as the light spectrum. Each of these parameters may distinctly affect a susceptible animal, depending on the species’ photoreceptive properties and sensitivity. The reported negative impacts of ALAN on the natural behavior of various animals include sleep disruption (Bedrosian et al., 2013; Raap et al., 2015), changes in foraging activity and predation (Sanders et al., 2018; Sanders et al., 2021), loss of spatial orientation (Eisenbeis, 2006; Foster et al., 2021), and disturbance to temporal partitioning and synchronization of activity (Amichai and Kronfeld-Schor, 2019; Levy et al., 2021).

Crickets (Gryllidae) have been used as insect models for behavioral research, neuroethology and, specifically, studies of circadian activity (Huber et al., 1989; Tomioka and Chiba, 1989; Horch et al., 2017). The nocturnal field cricket *Gryllus bimaculatus* expresses temporal shifts in its locomotor activity, stridulatory activity, or circadian gene expression following exposure to changes in illumination patterns (Loher, 1972; Loher, 1989; Abe et al., 1997; Uryu, 2014; Kutaragi et al., 2018; Tomioka and Matsumoto, 2019). Moreover, in constant darkness the compound eyes of *G. bimaculatus* display a clear circadian rhythm in their response to stimuli, with a diurnal minimum and a nocturnal maximum (Tomioka and Chiba, 1982), as well as a circadian sensitivity of visual interneurons and serotonin levels (Tomioka et al., 1993). We recently demonstrated the negative impact of ALAN on male field crickets (Levy et al., 2021). Lifelong exposure to even dim ALAN intensity had a desynchronizing effect on the crickets’ stridulation and locomotion behaviors. Recently, we also found tissue-, genes- and light-intensity-dependent changes in circadian gene expression in various body parts of the cricket, including the brain and optic lobes (Levy et al., 2022).

Here we studied the effects of exposing male *G. bimaculatus* to a pulse of ALAN of different intensities on stridulation and locomotion behavior. We further investigated the effects of a transition from light-dark conditions to constant light on the two behaviors in the same individual. We describe a light-intensity-dependent behavioral reaction, presenting simultaneous changes in distinct activities’ levels, or simultaneous negative and positive masking effects on stridulation and locomotion behavior, respectively. We also demonstrate light-intensity-dependent changes in the period of daily activity rhythms following transition from light:dark (LD) to light:ALAN (LA) or constant light (LL) treatments. The findings from this work emphasize the importance of utilizing a multi-behavioral approach in order to obtain a more complete understanding of the impact of ALAN, and its related mechanisms.

## 2 Materials and methods

### 2.1 Cricket rearing conditions

*G. bimaculatus* crickets were reared in plastic containers equipped with an egg carton shelter under a constant temperature of 26°C ± 1°C and a 12 h light:12 h dark (LD) cycle [compact fluorescent light (CFL, 40W, NeptOn, 6500K)]. Daylight intensities measured above the containers ranged from 250 to 350 lux, while the actual daylight intensities under the shelter ranged from 20 to 60 lux. The crickets were fed three times a week with dog-food pellets and vegetables. The rearing boxes contained water flasks with absorbent cotton wool.

### 2.2 Experimental set-up

**FIGURE 1:**
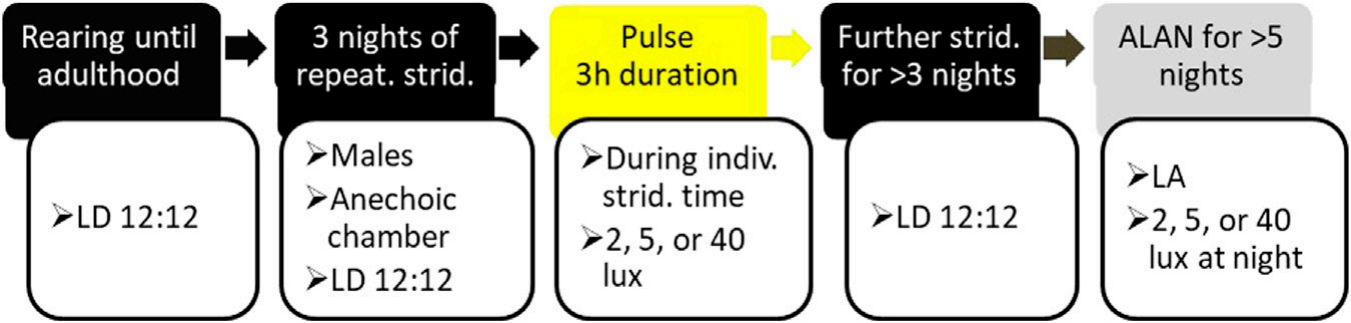
The experimental timeline: Adult, male crickets reared under LD 12:12 illumination regime (see Materials and Methods for details) were placed individually, each in an anechoic chamber, and their stridulation and locomotion monitored simultaneously (two left black boxes). On the night following three consecutive nights of consistent stridulation behavior, the experimental crickets were exposed to a light pulse (yellow box) of the duration of three hours and an intensity of 2, 5, or 40 lux. Following three to five additional nights under LD regime (right black box), the experimental animals were subjected to 12 h light:12 h ALAN (LA) or constant light (LL) conditions for at least five consecutive days and nights (grey box).

The experimental methods largely followed those described in Levy et al. (2021). In brief, the crickets were maintained individually in custom-made experimental anechoic chambers, preventing intraspecific communication while enabling continuous and simultaneous monitoring of stridulation and locomotion behaviors (see Levy et al., 2021; Figure 1 therein). Stridulation was recorded using a condenser microphone, an amplifier, a computer, and RavenLite2.0.0. Locomotion activity was captured from above by an infra-red (IR) surveillance camera, using motion detection (Ballon, 2015). Illumination was provided using a 5W CFL bulb (NeptOn, 6500 K, Supplementary Figure S1), emitting 40 lux, while lower illumination intensity was obtained by shading the light bulb.

### 2.3 Experimental procedure

Adult males 3–7 days post adult emergence were removed from the breeding colony and placed in the anechoic chamber under similar LD conditions as in the colony (for experimental timeline, see Figure 1). Stridulation and locomotion were monitored simultaneously throughout the experiment. The crickets were monitored until reaching three consecutive nights during which consistent stridulation behavior was demonstrated. On the following night, during the same time as that of the preceding consistent stridulation, the experimental cricket was exposed to a 3-h duration light pulse at an intensity of either 2, 5, or 40 lux (Figure 1). The timing of consistent stridulation varied between individuals, and, consequently, also did the precise *Zeitgeber* time of the pulse. In cases where no consistent behavior could be detected over the course of a ten-night period, the experimental crickets were replaced by new males, until the experimental pre-conditions were reached. Following at least three additional nights under the same LD regime, the experimental animals were subjected to one of several treatments: 24 h cycles of 12 h 40-lux light: 12 h 0-lux dark (LD), 12 h 40-lux light: 12 h 2-lux light (LA2), 12 h 40-lux light: 12 h 5-lux light (LA5), 12 h 40-lux light: 12 h 40-lux light (LL). For each cricket, the continuous night-long ALAN was of the same intensity as that used for the pulse.

### 2.4 Data processing and statistical analysis

Stridulation data extraction and processing followed Levy et al. (2021), using “R”, version 3.4.1 (R Core Team, 2020), the “Rraven” open source package (Araya-Salas, 2017), and RavenPro1.5 (Bioacoustics Research Program, 2013). Data processing and statistical analyses were conducted in PYTHON v. 3.7 (PyCharm, JetBrains), SPSS version 21 (IBM Corp., Armonk, NY, United States) and PRISM 8 (GraphPad Software, San Diego, CA, United States). The number of stridulation syllables and locomotion events were assessed per animal in 10 min bouts. Values were normalized for each individual by dividing that individual’s values by its own maximum value, resulting in an activity index ranging from 0 to 1 (no activity to maximum activity). Stridulation and locomotion during the exact same hours of the pulse in the night preceding and following the pulse manipulation were averaged and compared to those of the time of the pulse. Stridulation behavior was analyzed using a one-tailed Repeated Measures ANOVA with Tukey’s multiple comparisons, while locomotion behavior was analyzed using a one-tailed non-parametric Friedman test with Dunn’s multiple comparisons. Stridulation and locomotion behaviors were also assessed and compared one hour before the pulse, one hour during the pulse, and one hour after the pulse on the exact same night of the pulse manipulation, using a one-tailed Wilcoxon Signed Ranks Test.

Periodogram analyses of the activity rhythm periods were performed using the ImageJ plugin ActogramJ (Schmid et al., 2011). Comparisons of periodogram analyses of the activity rhythm periods before and after LA or LL, as well as the changes within treatment, the absolute differences between both behaviors, and the quantitative analyses were performed using the Kruskal–Wallis test, followed by a Dunn Sidak correction. Spearman’s rank-order correlation was used to evaluate the relationship between stridulation and locomotion behavior, as well as between the absolute behavioral reaction to the pulse and to the transition to LA or LL for each behavior. Combined actograms of stridulation and locomotion behavior were created using a custom-written code in “R”, version 3.4.1. (Supplementary Material).

## 3 Results

### 3.1 Behavioral, temporal patterns under LD conditions

**FIGURE 2:**
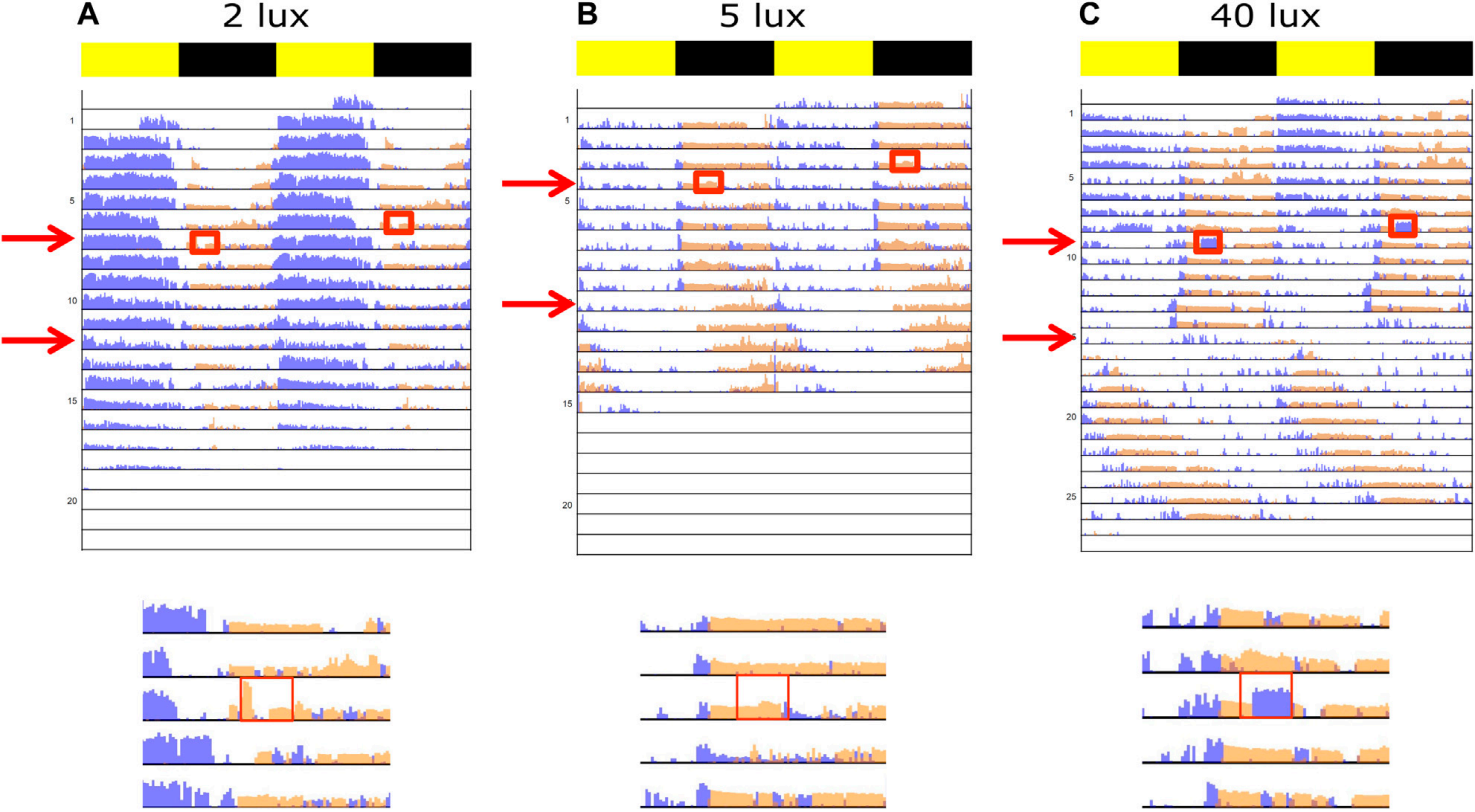
Double-plotted actograms representing light-pulse-dependent behavior of experimental crickets. Normalized activity of stridulation (orange) and locomotion (blue) are shown for each light intensity **(A)** 2 lux, **(B)** 5 lux, and **(C)** 40 lux. The first arrow and red rectangle represent the pulse treatment showing either a weak to no behavioral reaction **(A, B)**, or a strong masking reaction to the light pulse **(C)**. The general area of the plot around the time of the pulse was enlarged. The second arrow indicates the transition from LD into LA (constant, night-long ALAN) or LL (constant 40 lux), whereas nocturnal light intensity matched the pulse intensity. Yellow and black bars indicate diurnal and nocturnal phases, respectively.

Confirming our previous report (Levy et al., 2021), the experimental crickets exposed to control LD conditions exhibited a synchronized activity rhythm of 24 h, with stridulation being nocturnal and locomotion activity mainly diurnal (Figure 2, top rows in all the panels).

### 3.2 A light pulse at night evoked simultaneous negative and positive changes in specific activities’ levels

**FIGURE 3:**
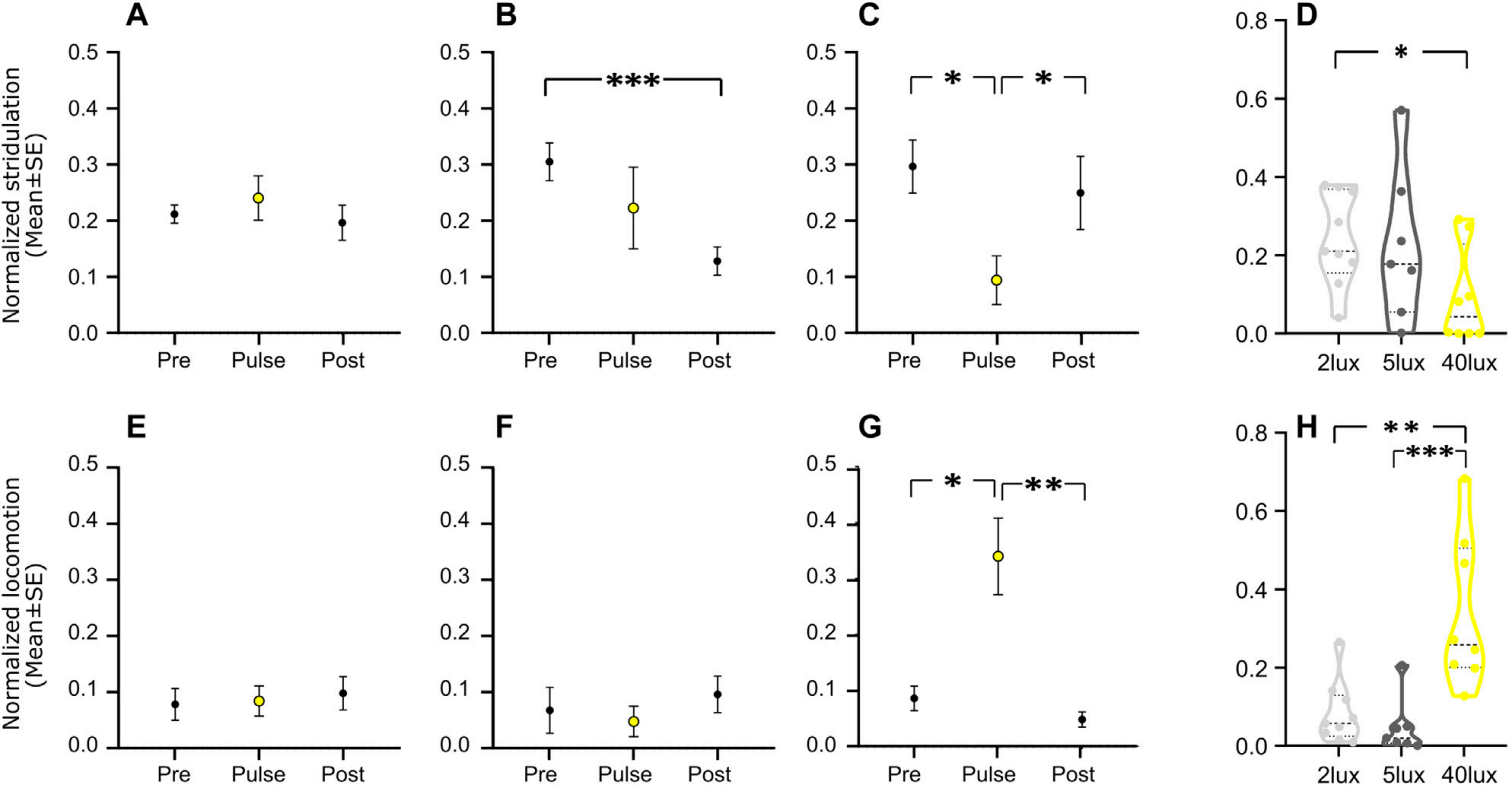
Normalized mean level (±s.e.) of stridulation **(A–C)** and locomotion **(E–G)** behavior, three nights before (‘Pre’), during the pulse treatment (‘Pulse’, yellow), and three nights after (‘Post’) a pulse of 2 lux (**A**, **E**; *n* = 9), 5 lux (**B**, **F**; *n* = 8), and 40 lux (**C**, **G**; *n* = 8). **(D–H)** The normalized mean behavior levels (**D**, stridulation; and **H**, locomotion) during the pulse treatment under the different pulse intensities. *p < 0.05, **p < 0.01, ***p < 0.001.

The effect of a light pulse on the experimental crickets was largely intensity dependent (Figure 2): whereas a behavioral reaction was detected in the 2 lux and 5 lux treatments in only a small number of cases (Figures 3A, B, E, F), a clear simultaneous negative (for stridulation) and positive (for locomotion) masking effect was observed at 40 lux (Figures 2C, 3C, G). Stridulation activity significantly dropped during the light pulse, compared to that on the day prior to the pulse (Repeated Measures ANOVA with Tukey multiple comparisons, F_7,7_ = 4.257, *p* = 0.023, *n* = 8, Figure 3C), while locomotion activity increased (Friedman test with Dunn’s multiple comparisons, χ2(2) = 9.750, *p* = 0.037, *n* = 8, Figure 3G). The level of stridulation and locomotion activity during the light pulse was found to be affected by the light intensity (stridulation: between 2 and 40 lux; Kruskal–Wallis with Dunn’s multiple comparisons, χ_2,24_ = 5.304, *p* = 0.024, and for locomotion: between 2 and 40 lux, and between 5 and 40 lux; χ_2,24_ = 13.41, *p* < 0.01 Figures 3D, H, respectively). While not all individuals seemed to be affected by the pulse, the percentage of animals presenting a clear effect increased with higher light intensity. The pulse seemed to have no residual effect on behavior during the following night: in the 40 lux treatment, activity during the night of the pulse and the night following the pulse differed significantly for both stridulation and locomotion behavior (Repeated Measures ANOVA with Tukey multiple comparisons, F_7,7_ = 3.609, *p* = 0.043, *n* = 8, 1-tailed; Friedman test with Dunn’s multiple comparisons, χ2(2) = 9.750, *p* = 0.004, *n* = 8, 1-tailed, Figures 3C, G, respectively). A control group (no pulse treatment) revealed no differences between days (during the duration of the experiments), excluding possible time-dependent differences between the pre-pulse and post-pulse conditions (data not shown). Pre- and post-pulse behavior significantly differed only in the 5 lux stridulation group (Repeated Measures ANOVA, F_7,7_ = 9.084, *p* < 0.001, *n* = 8, Figure 3B).

**FIGURE 4:**
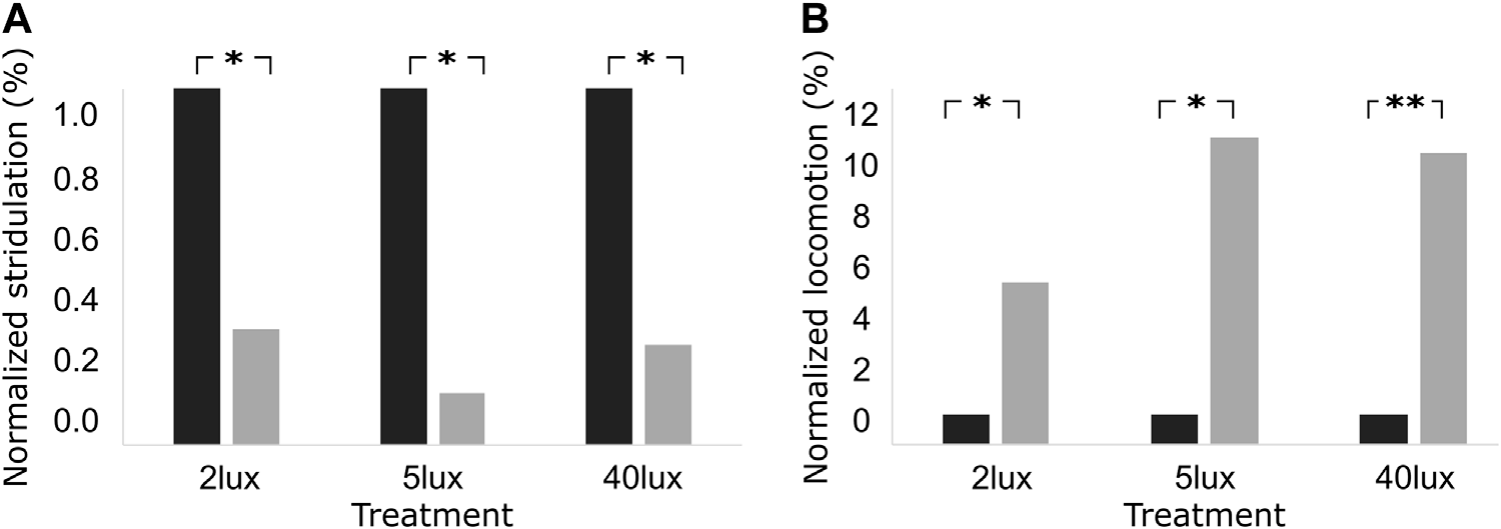
Percentage of normalized level of **(A)** stridulation, and **(B)** locomotion behavior, one hour before (black), and one hour after (grey) the light pulse treatment of 2 lux, 5 lux, and 40 lux. *p < 0.05, **p < 0.01, representing one-tailed *p*-values.

Despite of the general absence of behavioral changes on the night following the night of the pulse, we further explored the possibility of pulse-induced effects exceeding the limited period of the pulse duration itself. We therefore compared the behavior of the crickets one hour immediately prior to the pulse treatment, and one hour immediately after the pulse treatment. Figure 4 reveals an overall consistent decrease in stridulation (Figure 4A) and increase in locomotion (Figure 4B) activity during the hour following the pulse termination. The effect was significant for all experimental groups (light intensities) for both observed behaviors (one-tailed Wilcoxon signed ranks test; *p* < 0.05 for all). The light pulse, thus also induced some residual behavioral effect (Figure 4); even when this had been insignificant during the pulse itself (as in the case of the 2 and 5 lux treatments, Figure 3).

### 3.3 Transition to LA/LL evoked changes in temporal patterns of stridulation and locomotion behaviors

**FIGURE 5:**
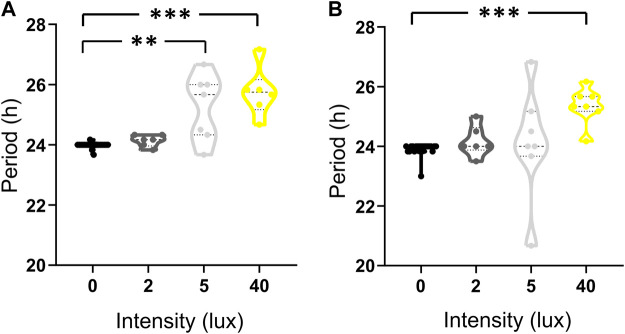
The transition from LD to LA or LL affects the individual daily activity periods of stridulation **(A)** and locomotion **(B)** in a light intensity dependent manner. LD (before transition, black, *n* = 20), LA_2_ lux (dark grey, *n* = 7), LA_5_ lux (light grey, *n* = 7) and LL_40_ lux (yellow, *n* = 6, *n* = 7 for stridulation and locomotion, respectively) treatments. *p < 0.05, **p < 0.01, ***p < 0.001.

A transition of the experimental conditions from LD to LA and LL affected the behavior of all individuals, as reflected in the high percentage of induced free-run behavior (Figure 5). This differed between the stridulation and locomotion behaviors (94.4%, and 68.75%, respectively; *n* = 20). For both behaviors, however, the period of the behavioral cycle was found to be light-intensity-dependent (Figure 5): the median period of stridulation behavior increased in a light-intensity-dependent manner, while the median of locomotion behavior increased only at 40 lux (Table 1). The period of stridulation under LD conditions differed significantly from that of the 5 and 40 lux treatments (Kruskal–Wallis with Dunn’s *post hoc* test, *p* < 0.005, and *p* < 0.001, respectively, Figure 5A); while for locomotion behavior, only the 40 lux treatment significantly differed from that of LD (Kruskal–Wallis with Dunn’s *post hoc* test, *p* < 0.001, Figure 5B). Thus, even an ALAN of 2 lux leads to free running rhythms of stridulation.

**TABLE 1:** The median values of stridulation and locomotion daily activity cycle periods in the experimental groups under LD (0 lux, *n* = 20), LA of 2 lux (*n* = 6), 5 lux (*n* = 7), and 40 lux (LL, *n* = 6, *n* = 7 for stridulation and locomotion behavior, respectively).

	0 lux	2 lux	5 lux	40 lux
Stridulation	24.00	24.17	25.67	25.75
Locomotion	24.00	24.00	24.00	25.33

A quantitative comparison of the overall normalized diurnal and nocturnal stridulation activity levels prior to and following the transition to LA and LL revealed a significant change in the diurnal 5 lux and higher activity under 40 lux in both diurnal and nocturnal stridulation (*p* < 0.05 for all, Kruskal–Wallis test with Dunn’s multiple comparisons). However, no significant quantitative differences were detected in locomotion behavior (*p* > 0.05 for all, Kruskal–Wallis test). Moreover, no correlation was found between the calculated LA or LL activity periods of stridulation and locomotion (Spearman’s rank-order correlation, r (12) = 0.30, *p* = 0.29), emphasizing the differential impacts of the treatment on both examined behaviors and its effect on de-synchronization among the two behavioral rhythms.

### 3.4 No correlation was found between the responses to the pulse and to the transition to LA/LL

To investigate a probable connection between stridulation and locomotion behavior, as well as a possible individual susceptibility to ALAN intensity, the correlation among the different behavioral parameters were calculated. No correlation was found between the absolute effect of the pulse treatment and the absolute change in activity periods following transition to LA or LL (Spearman’s rank-order correlation, r (12) = −0.26, *p* = 0.36; and r (12) = 0.39, *p* = 0.17 for stridulation and locomotion, respectively).

## 4 Discussion

In this study, in order to provide novel insights into the adverse effects of ALAN and into the mechanisms of these effects on animal behavior, we investigated the impact of a pulse of light at night on stridulation and locomotion behavior in the field cricket *G. bimaculatus.*

Under exposure to 40 lux we identified simultaneous changes in specific activity levels in the same individual crickets; simultaneous negative and positive masking effects on the cricket’s stridulation and locomotion behavior, respectively. Such findings, to the best of our knowledge, have never been described in an insect. The masking phenomenon reflects a flexible, immediate reaction to a stimulus without affecting the animal’s endogenous rhythm (Kuhlman et al., 2018). Aschoff (1960) suggested that light increases diurnal activity in light-active animals and decreases it in dark-active animals. In accord with the Aschoff’s rule, the light pulse evoked a negative masking effect in the crickets’ nocturnal behavior, reflected in a decrease and even cessation of stridulation; while the diurnal behavior (locomotion) increased (positive masking) under the light pulse. Similarly, in rodents, exposure to ALAN was reported to result in negative masking in a nocturnal rodent, while no effect was observed in a diurnal rodent (Rotics et al., 2011). In addition, the presented increase in crickets’ diurnal locomotion activity during the light pulse is in agreement with the previously described increase in burst and intensity of locomotion during a pulse of light (Germ and Tomioka, 1998; though this was demonstrated under constant darkness). The inter-individual variations and light-intensity dependent percentage of crickets presenting a masking response in the current work (as seen in Figure 3), may indicate both an individual and a population-related illumination-sensitivity threshold.

It should be noted, that we had a preference in the current study to present the ALAN-pulse 1–2 h after lights off, as the early and late subjective night were reported to be more affected by a pulse of light (Kutaragi et al., 2016). However, the pulse was not submitted at a specific *Zeitgeber* time, but rather at the individual’s timing of previous consistent stridulation. These differences may have affected the responsiveness of the experimental individuals, leading to an increased variance.

The reactions of animals to different light stimuli are affected by many factors, such as the species’ way of life (i.e., nocturnal, diurnal; Helm et al., 2017; Kronfeld-Schor and Dayan, 2003; Mrosovsky, 1999), the specific properties of the animal’s visual system (Warrant and Nilsson, 2006; Land and Nilsson, 2012), visual sensory processing (Blum and Labhart, 2000; Okamoto et al., 2001; Mappes and Homberg, 2004), and of course the nature of the specific stimuli. *G. bimaculatus* is a nocturnal species, which possesses three types of visual receptors: UV (peak: 332 nm), blue (peak: 445 nm) and green (peak: 515 nm) (Zufall et al., 1989), and whose visual system is adapted for signal processing in dim light (Zufall et al., 1989; Sakura et al., 2003; Frolov et al., 2014). While the blue receptor has been reported to be responsible for polarization vision (Labhart et al., 1984; Herzmann and Labhart, 1989), the green-sensitive opsin-long wavelength (*OpLW*) was described to be the major circadian photoreceptor molecule, responsible for photic entrainment (Komada et al., 2015). Both, the visual and the circadian pathways start at the compound eye. Information from the eye is then transmitted to the optic lobe, where the circadian clock is assumed to reside, and processed in parts of the lamina, medulla and lobula of the optic lobe (Tomioka and Chiba, 1982; Tomioka and Chiba, 1985; Tomioka and Chiba, 1989; Tomioka and Chiba, 1992).

Insects, such as crickets, locusts, and cockroaches, may have several parallel neuronal pathways reaching from the compound eye to the brain, serving for similar or different functions (Okamoto et al., 2001; Homberg et al., 2003; Homberg et al., 2011; Homberg, 2004; Helfrich-Förster, 2020). Whether masking and entrainment use the same neuronal pathway or different ones, is still far from fully resolved. However, the current findings together with available other data suggest a common pathway for both: here we induced masking by a single light pulse, shown previously to affect circadian clock related transcriptional responses in the cricket one hour following the pulse (Levy et al., 2022). Our observations also revealed some residual effects of the pulse, expressed in a change in the percentage of normalized behavioral level one hour after pulse termination. This persisted even under light intensities for which the pulse itself seemed to have no effect. Such a lingering effect has not been described for rodents (Rotics et al., 2011). In a previous study on crickets, repetitive 15 min pulses (at 4, or 8 h intervals) under constant darkness evoked rhythm synchronization to the timing of the light pulses (Germ and Tomioka, 1998). It is therefore probable that in addition to the observed transient change (masking), a 3 h pulse of light at night triggers additional effects, which may be involved in the observed residual changes and maybe also in the entrainment to repetitive stimuli. These results are in line with Aschoff (1960) who suggested a connection between masking and entrainment, which may depend on the frequency and intensity of the stimuli.

The transition to LA or LL evoked a reaction in at least one of the two tested behaviors in all the experimental crickets, including in individuals that seemed unaffected by the earlier pulse. Our findings suggest, therefore, a light-intensity as well as a light-duration-dependent effect of ALAN on cricket behavior. This is in agreement with Germ and Tomioka’s (1998) findings, showing that in crickets frequent light pulses modulated the free-running period, depending on the interval of the pulses. The herein described differences in the median of the period of stridulation and locomotion activity patterns are in agreement with our previous experimental findings regarding lifelong exposure to ALAN illumination (Levy et al., 2021). These differences and the lack of correlation among the two behaviors could indicate a different susceptibility of stridulation and locomotion behaviors to the same light signals. It may also be that the differences reflect a more pronounced effect on the nocturnal than the diurnal behavior, simply since the light signal was presented at night. Our findings indicate no correlation between the stridulation and locomotion activity periods. No correlation was also found between the change during the pulse treatment and the LA/LL-induced change in activity periods for both behaviors. These findings are in accord with those of Levy et al. (2021), suggesting that the desynchronization of stridulation and locomotion activity rhythms reflect control by several peripheral clocks. We suggest that these different results for stridulation and locomotion indicate that the crickets’ behavioral reactions to light may rely on several mechanisms, rather than just one, but remain unclear to date and require further study.

Interestingly, in our previous study (Levy et al., 2021), lifelong illumination patterns evoked a high percentage of arrhythmic stridulation and locomotion behavior (29%, and 42.8%, respectively. In contrast, here, only one individual (14%) subjected to 40 lux (LL) presented arrhythmic behavior. This may be due to one or more of several reasons: First, the complexity of the experiments described in the current report have resulted in a relative small sample size, and it may be possible that a larger sample size would have included some additional arrhythmic individuals. Second, the experiments were conducted only with crickets which demonstrated consistent stridulation behavior during three consecutive nights. This pre-requisite may have filtered out individuals with a stronger tendency for arrhythmicity. Third, the crickets in (Levy et al., 2021) were submitted to lifelong ALAN, whereas the crickets in this study were raised under LD and transferred to ALAN as adults. It may be that the exposure to lifelong ALAN resulted in more arrhythmic crickets.

In summary, to date the response to a light pulse has been mostly studied in relation to phase shifts or entrainment under constant darkness. Here, we present instantaneous masking effects in two key insect behaviors. We describe for the first time a simultaneous pulse-induced negative and positive reaction, as well as a residual effect. We also demonstrate a change in behavior generated by transition to LA or LL conditions. This study and its findings add to the alarming effects of ALAN on living organisms, both individuals and populations. Our findings indicate the importance of darkness for timekeeping in nocturnal insects, and present the harsh, and fast effects of a pulse of light or chronic dim-ALAN on activity rhythms and courtship behavior of the crickets. The findings indicate that not only can life-long or night-long exposure to ALAN alter animal behavior (Rich and Longcore, 2006; Levy et al., 2021; Borges, 2022; Dominoni et al., 2022), but so too can a transient light exposure, such as a single pulse of ALAN (Rotics et al., 2011; Spoelstra et al., 2018; Levy et al., 2022). This aspect should be added to the other possible effects of ALAN, as it may become a stressor when frequently experienced, or even entrain individual behaviors, as shown for the cricket (Germ and Tomioka, 1998). The findings from this study provide further new insights into the importance of a multi-modal approach in order to more fully uncover the effects of ALAN on animal behavior and populations.

## Data Availability

The original contributions presented in the study are included in the article/Supplementary Materials, further inquiries can be directed to the corresponding author.

## Supplementary material

SUPPLEMENTARY FIGURE S1 Spectrograms of the light bulb used in the anechoic chamber for all experimental groups (LD, LA2, LA5, and LL). **(A)** 40 lux, **(B)** 5 lux, **(C)** 2 lux. The light spectra were recorded using the Sekonic Sprectromaster C-700 (North White Plans, NY, United States). https://doi.org/10.6084/m9.figshare.22060328.v1 for the code for the actograms Levy, Keren (2023): Actogram two behaviors code. figshare. Journal contribution.
