# Supplementary figures and images for: Exposure to a nocturnal light pulse simultaneously and differentially affects stridulation and locomotion behaviors in crickets
Source: Front Physiol. 2023 Mar 16;14:1151570. doi: 10.3389/fphys.2023.1151570 (PMC10061070; doi:10.3389/fphys.2023.1151570)

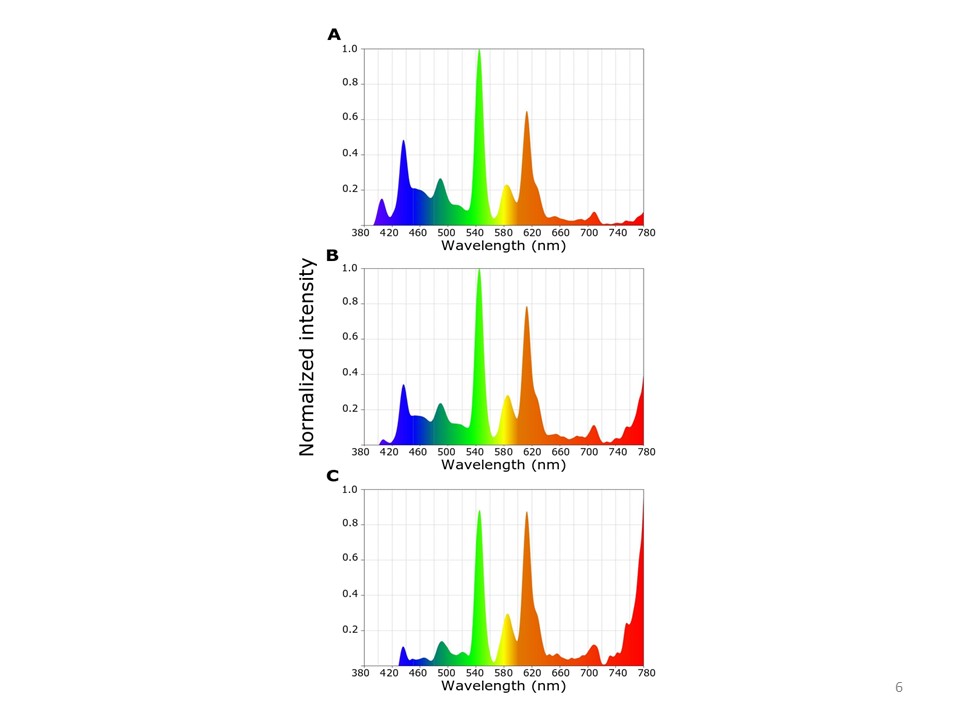

Supplement: Supplementary file 1 [file Image1.jpg]
